# A clinical series using intensive neurorehabilitation to promote functional motor and cognitive skills in three girls with *CASK* mutation

**DOI:** 10.1186/s13104-017-3065-z

**Published:** 2017-12-19

**Authors:** Stephanie C. DeLuca, Dory A. Wallace, Mary Rebekah Trucks, Konark Mukherjee

**Affiliations:** 1 Virginia Tech Carilion Research Institute, Neuromotor Clinic 2 Riverside Circle, Roanoke, VA 24016 USA; 2 Department of Pediatrics, Virginia Tech Carilion School of Medicine, Roanoke, VA USA; 3 Rehabilitation Health and Wellness, Jefferson College of Health Sciences, Roanoke, VA USA; 4 School of Neuroscience & Department of Psychology, Virginia Tech, Blacksburg, VA USA; 5 Department of Psychiatry, Virginia Tech Carilion School of Medicine, Roanoke, VA USA; 6 Department of Biological Science, Virginia Tech, Blacksburg, VA USA

**Keywords:** Microcephaly, Intellectual disability, Neurorehabilitation, Neuroplasticity

## Abstract

**Objectives:**

Children with microcephaly face lifelong psychomotor, cognitive, and communications skills disabilities. Etiology of microcephaly is heterogeneous but presentation often includes seizures, hypotonia, ataxia, stereotypic movements, attention deficits, excitability, cognitive delays, and poor communication skills. Molecular diagnostics have outpaced available interventions and most children receive generic physical, speech, and occupational therapies with little attention to the efficacy of such treatments. Mutations in the X-linked intellectual disability gene (XLID) *CASK* is one etiology associated with microcephaly which produces mental retardation and microcephaly with pontine and cerebellar hypoplasia (MICPCH; OMIM# 300749). We pilot-tested an intensive therapy in three girls with heterozygous mutation in the gene *CASK* and MICPCH. Child A = 54 months; Child B = 89 months; and Child C = 24 months received a targeted treatment to improve gross/fine motor skills, visual-motor coordination, social interaction, and communication. Treatment was 4 h each weekday for 10 treatment days. Operant training promoted/refined goal-directed activities. The Peabody Developmental Motor Scales 2 was administered pre- and post-treatment.

**Results:**

Child A gained 14 developmental months; Child B gained 20 developmental months; and Child C gained 39 developmental months. This case series suggests that children with MICPCH are responsive to intensive therapy aimed at increasing functional skills/independence.

*Trial Registration* ClinicalTrials.gov Registration Number: NCT03325946; Release Date: October 30, 2017

## Introduction

Microcephaly refers to a neurodevelopmental condition associated with architecturally normal but smaller brain [1–8]. The classic and most recognized sign of microcephaly is that the occipito-frontal head circumference falls below that of age-matched peers. This difference can be more than three standard deviations of the typical head circumference [1–8], and is usually recognized during the first year of life. Clinical outcomes range from individuals being asymptomatic to presenting with profound cognitive and psychomotor disabilities or even refractory seizures and lethality [1–8]. The reduced brain size is often associated with decreases in neural capacity and neurological deficits that manifest via a myriad of developmental delays and subsequent long-term impairments in intellectual abilities, cognitive processing, gross/fine motor skills, visual/motor coordination, and speech production. Additionally, individuals may have altered sleep patterns, hypotonia, stereotypic movements, attention deficit, and excitability [1–8]. In general, the presentation of signs and symptoms that individuals with microcephaly manifest are aligned with the severity of the neurological defect rather than an etiological cause [1–8]. Neurological deficits are not progressively degenerative in most cases of microcephaly, but signs and symptoms appear more pronounced as children age because they make less developmental progress and fall further and further behind age-matched peers.

Microcephaly can be primary (prenatal) resulting from genetic mutations or secondary (postnatal) acquired resulting from trauma, toxins, infections (e.g. Zika virus, Cytomegalovirus and Toxoplasma), or deprivation of maternal/child nutrition [3, 4, 6–9]. Different etiologies affect brain growth and development and some are associated with brain malformations such as lissencephaly and pontocerebellar hypoplasia [10–16]. Infants and children with microcephaly are referred for a variety of services and interventions with a goal of improving developmental trajectories, maximizing abilities, and positively impacting quality of life [6]. Formal investigations into rehabilitative interventions tend to ignore the underlying etiologies; even though, there is a large amount of data available from animal models that might inform treatment choices [16–18]. For example, animal models suggest that mutations in molecules that are known to impact plasticity are more likely to be less responsive to rote training [16–18], but may exhibit improvement with brain stimulation [19]. On the other hand, animal models that are secondary to environmental toxins show functional improvement in enriched environments alone [20, 21].

Mutations in the X-linked (XLID) gene *CASK* are associated with mental retardation and microcephaly with pontine and cerebellar hypoplasia (MICPCH; OMIM# 300749). This etiology disproportionately affects females because *CASK* is an X-linked essential gene [22] and is characterized by global cognitive, psychosocial and motor deficits [10, 16]. The motor development of these children is often delayed by years, and they remain well behind age-matched peers in intellectual and communication abilities, usually with profound speech production disabilities. *CASK* heterozygous knockout female mice (CASK^(+/−)^) phenocopy the human motor limitations with high fidelity [20, 21] showing motor incoordination and ataxia. Interestingly, they do display rapid motor learning on rotorod treadmill training [21].

Intensive bursts of neurorehabilitation have proven efficacious in successfully helping children with other neuromotor etiologies gain increased skills [23–28]. For example, intensive treatment protocols delivered by occupational and physical therapists trained in operant conditioning have consistently demonstrated the ability to help infants and children with Cerebral Palsy gain motor skills [23–28]. Based on the combined lines of evidence from animal models of microcephaly demonstrating positive responses to training and children with Cerebral Palsy benefiting from intensive rehabilitation, we hypothesized that an intensive burst of therapy could be useful in promoting skill acquisition in young children with MICPCH.

## Main text

### Methods

#### Study design

This case series involves three females with MICPCH due to *CASK* gene mutations [11]. The University’s Institutional Review Board approved the collection and use of data. Informed consent was obtained from each family prior to participation.

*Participants* All children displayed global developmental delays that presented with limitations in; fine/gross motor skills, speech production, communication, social interaction, and cognition. All children were receiving therapy but remained behind age-matched peers.

 (i) Child A (54 months of age) has a variant in *CASK* gene (NM_003688) c.2221 + 1G > C with microcephaly and mild hypoplasia of the pons and cerebellum. Her speech was non-responsive single-word productions (stereotyped and repetitive). If you requested that she identify an item from a set that had been verbally identified, she would choose the last item, consistently, even if it did not correspond correctly with the requested item. She was unable to choose colors, shapes, or animals, correctly, even though she could mimic the spoken names. She followed one-step directions and would mimic short-sequenced behaviors. For example, the participant could not stack blocks or copy basic shapes (e.g. drawing a circle) when directed, but she would turn the pages of a book after a therapist turned the pages of a book or crawl on hands and knees to follow a therapist. When ambulating, she would walk into items with little awareness of the items in her path. When behaviorally challenged, she would demonstrate emotional displays that were sporadic but included periods of calm and inattention. She would not participate in pretend play. Play-behaviors appeared impulsive.

 (ii) Child B (89 months of age) has p.Arg537Ter (CGA > TGA): c.1609 C > T in exon 17 in the *CASK* gene (NM_003688.3) with microcephaly, and the right cerebellar hemisphere is smaller than the left. Her speech was limited to single words or two-word combinations (marked by echolalia). She, too, chose the last items identified when a request was made. She was able to consistently identify colors and a few animals. She had decreased environmental awareness and would often run away from supervising adults. Her emotional outbursts were marked by crying episodes where she immediately sought parental care. She could use a marker to make a mark but could not draw basic shapes, letters or color within the lines of a picture. She could use scissors to cut paper but could not cut out shapes or across a line on a paper.

 (iii) Child C (24 months of age) has p.Gln36* c.106C > T (NM_003368.3) with microcephaly and hindbrain hypoplasia. She had no speech productions and presented with fine and gross motor delays. She was unable to sit independently for longer than 30 s and could not transition to sit in an age-typical manner (lying in a supine position she would attempt to come to sitting with full-body flexion). She was not crawling and when facilitated into four-point weight-bearing, she would activate full extension rather than maintain that position. She inconsistently could use a gross grasp for a few items but could not target a placement for release. She could not activate cause-and-effect toys.

*Treatment protocol* Treatment was delivered for 4 h each weekday for 10 days and focused on improving motor skills, social interaction, and communication skills. Therapy sessions were delivered via participation in play and daily living activities that were systematically shaped towards targeted tasks via operant conditioning [23–28]. Operant conditioning involves immediate and specific reinforcement of isolated skills that are then successively chained together towards a more complex or skillful behavior. Increased proficiency is required as skills develop. The process starts by reinforcing basic (sometimes-random) movements or behaviors which are reinforced when repeated. Specific refinement requests are made, and across time increased proficiency in the targeted behavior is required to obtain the reinforcement. All treatment activities were tailored to each child’s skill and developmental age. All children were asked to make targeted speech productions in response to requests. For the youngest child this included beginning consonant sounds and sign language.

*Assessments* Children were assessed with the Peabody Developmental Motor Scales 2 (PDMS) [29] prior to and immediately after the protocol. This measure examines development and is comprised of 6 subtests (i.e., reflexes, stationary, locomotion, object manipulation, grasping, visual-motor integration). Five of the 6 subtests result is three developmental quotients; a gross motor quotient (GMQ) includes the stationary, and locomotion subtests; a fine motor quotient (FMQ) includes the grasping, object-manipulation, and visual-motor integration subtests; and a total motor quotient (TMQ), which includes all 5 of these subtests. The reflexes subtest is only for children under 12 months of age and was not used. The three developmental quotients are known to have good test–retest reliability in older children; GMQ r = .93, FMQ r = .94, and TMQ r = .96.

The PDMS is norm-referenced for children between birth and 5 years of age. Child B was older than 5 years, but fell below this age in the developmental skills targeted for treatment. Her scores were based on the tables associated with the highest age tables of the assessment (66–71 months). Use of this measure allowed comparison across all 3 children. Child C was also tested with the Gross Motor Functional Measure (GMFM) 88 [30]. The GMFM 88 has greater breadth of gross-motor items and is designed for children with motor limitations. Notes, videotapes, and observations made by parents were also used as descriptors of changes.

### Results

Tables 1 gives a qualitative summary of each child’s improvements. Changes in the PDMS 2 and the GMFM are shown in Table 2.

**Table 1:** Qualitative clinical improvement

Participant A	Participant B	Participant C
Speech changes		
*Production of multiple two to three word combinations and sometimes short sentences* *‘yellow dog’*	*Production of multiple word combinations and sometimes short sentences that included noun*–*verb*-*noun placement or noun*–*verb*-*adjective*-*noun placement*	*Production of one sign with consonant sounds* *Began signing “more”.\* *Consonant sounds of “B”,* *“M”, and “D”*
*Developed reciprocal speech with family. Asked for a food item by saying ‘I want (food item)’. The parent reported that this was the first time the participant had ever made an identifiable and specific request. Prior to treatment the participant would have simply reached to obtain the item*	*Developed conversational speech patterns. In response to the parent asking, if she wanted to go shopping, Participant B responded “I want one, two, three, shirts”*	*Increased production of verbalizations in response to activity and interaction*
	*Spontaneous signing with a song on the radio.*	
Social awareness changes		
Began to follow directions up to 4 steps	Began to follow directions up to 4–6 steps. ‘Get the markers and the paper, place them on the table and then draw a circle.’	*Following 1 step directions* *“Put the ball in”* *More intricate play: knew she had to put a ball in and then push the cause and effect toy to make it go*
Increased eye-contact.	Increased responsiveness to adult supervisors in unfamiliar environments. This included staying next to an adult with only verbal cues without running away, spontaneously	*Increase in independent play without constant adult interaction*
*Use of social greetings such as ‘hi’, ‘bye’, ‘thank you’, and ‘you are welcome’*	Decrease in emotional outbursts	
Began to avoid obstacles		
Cognitive & motor skill changes		
Increased object identification and delineation. ‘find the yellow flower and then the blue flower’	Increased object identification and delineation. ‘find the man with the policeman hat and then find the man with the fireman’s boots’.	Increased sitting balance
Less time required for correct object identification. (can we put numbers?)	Less time required for correct object identification. (can we put numbers?)	*Maintenance of 4*-*point weight*-*bearing*
*Could draw a circle and a cross*	*Could draw several shapes and a few letters*	*Transitioning to 4*-*point weight*-*bearing*
Better orientation of and placement of puzzle pieces	Better orientation of and placement of puzzle pieces	*Pull to knees on surface with stability to play with toy*
*Could stack blocks*	*Could use scissors to cutout basic shapes*	

First-time behaviors are italicized

**Table 2:** Standardized outcome measures

PDMS 2	Child A			Child B			Child C		
	Pre	Post	Change	Pre	Post	Change	Pre	Post	Change
Gross motor quotient	72	76	+ 4	102	104	+ 2	53	64	+ 11
Fine motor quotient	58	55	− 3	82	91	+ 9	52	73	+ 21
Total motor quotient	63	64	+ 1	93	97	+ 4	48	64	+ 14
GMFM-88	NT	NT		NT	NT		21	44	+ 23

*NT* not tested

**Fig. 1:**
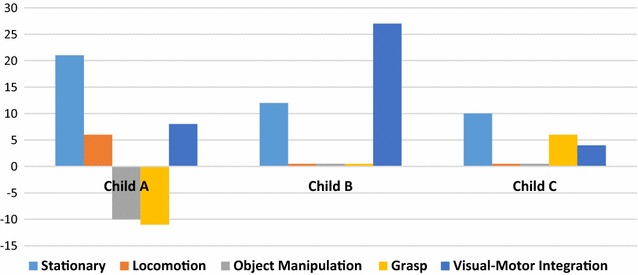
PDMS raw score changes by subtest

Figure 1 shows the change in raw scores for each child via each subtests of the PDMS. Two subtests changed across all 3 children. Paired sample *t* test indicate that the only subtest or developmental quotient that was statistically significant pre to post treatment across the three children was the stationary subtest with a t = 12.12, p = .007.

Raw scores were converted to age-equivalents for the PDMS 2, and the number of developmental months each child changed during the therapy period was; 14 months for Child A, 39 months for Child B, and 20 months for Child C. The oldest child had the largest gains in developmental months, which appears to be associated largely with increased visual-motor integration skills. The youngest child had the largest gain in the total motor quotient, which may represent the large gains in gross motor skills on both the PDMS 2 and the GMFM.

### Discussion

Recent reports place the incidence of microcephaly as high as 12 cases per every 10,000 births [4, 30]. The traditional medical management of microcephaly usually focuses on short-term therapies with low dosage of one or 2 h a week, but there is little to no evidence in support of their efficacy [1, 2, 4–6, 8]. Our intensive treatment protocol demonstrated large increases in developmental progress across three females with a specific microcephalic etiology (i.e., MICHCP). Two questions come to mind. (1) What are the potential mechanisms for improvement? And, (2) can we extend our observations to other causes of microcephaly?

Given that animal studies indicate training may serve as a trigger for neurogenesis [17, 18], it is intriguing to consider that intensive neurorehabilitation may trigger neurogenesis in children with microcephaly. This case-series cannot directly address this question because only functional measures were obtained, but many of the functional changes were large gains that are likely associated with some forms of central nervous system changes. The subtests on the PDMS that demonstrated improvements across all 3 children interrogate complex motor issues that are greatly influenced by a child’s abilities to complete motor-planning and multistep tasks that use perceptual processing skills and or that are complex eye-hand coordination activities. Three of five subtests on the PDMS did not change for two children, and a third showed some declines, but all children demonstrated positive overall changes in performance. While the declines for the 1 child need to be considered, thoughtfully, the large overall gains are encouraging. These findings serve, primarily, as a ‘proof of principle’ for testing intensive therapies for children with MICPCH, but future randomized controlled studies need to better understand the possible impact of intensive therapy bursts on children with other microcephalic etiologies.

### Conclusions

Our findings suggest that intensive therapies can positively influence children with microcephaly by improving their skills and abilities. The treatment period used in this series was less than 1 month in total duration and the average number of developmental months gained across all 3 children was 24 months. Gaining 2 years of development in such a brief time might greatly alter each child’s long-term developmental trajectory.

### Limitations

The study design and small sample size require that interpretation of findings be viewed with caution. In addition, all children in the series were female, so we have no way to understand if findings might differ by gender. Lastly, the wide age span across the 3 participants is informative because all children positively responded, but age may play a key role in how efficacious intensive therapies are because of the increased brain plasticity in younger children. This needs to be addressed in future trials.

## Abbreviations

MICPCH: mental retardation and microcephaly with pontine and cerebellar hypoplasia
XLID: X-linked intellectual disability
PDMS: Peabody Developmental Motor Scales 2
GMQ: gross motor quotient
FMQ: fine motor quotient
TMQ: total motor quotient
GMFM: gross motor functional measure 88
